# Influence of egg traits on parasitism by *Trichogramma chilonis* Ishii, 1941 and *Telenomus remus* Nixon, 1937 against *Spodoptera frugiperda* (J.E. Smith, 1797)

**DOI:** 10.3389/finsc.2026.1749736

**Published:** 2026-02-17

**Authors:** Kushal Giri, Min Raj Pokhrel, Ghanashyam Bhandari

**Affiliations:** 1 Department of Entomology, Agriculture and Forestry University, Rampur, Bagmati, Nepal; 2 National Maize Research Program (NMRP), Nepal Agriculture Research Council (NARC), Rampur, Bagmati, Nepal

**Keywords:** fall armyworm, biocontrol agent, parasitism, egg scale covering, egg age

## Abstract

*Trichogramma chilonis* Ishii, 1941 and *Telenomus remus* Nixon, 1937 are the most common egg parasitoids of fall armyworm (FAW), *Spodoptera frugiperda* (J.E. Smith, 1797) in maize growing areas. FAW lays single to multilayered egg which are covered with degrees of scale thickness. Here, we assessed the parasitism of both parasitoids over different FAW egg densities (single layered), egg mass scale coverage (multilayered), and the egg ages. Two laboratory experiments were conducted from May to November 2022 under controlled conditions (24.3 ± 0.8 °C, 69.3 ± 2.2% RH). The first experiment, using a three-factor complete randomized design (CRD), involved two egg parasitoid species, single-layered eggs at three densities (20, 43, and 60 eggs), and three egg age groups (less than 12 hours, 24–36 hours, and 48–60 hours), each replicated three times. The second experiment involved two parasitoid species, multilayered eggs with three levels of egg scale coverage (fully covered, partially covered, uncovered), and three egg age groups, each replicated three times. *T. remus* exhibited significantly higher parasitism rates than *T. chilonis* in both single and multilayered egg masses. *T. remus* parasitized all egg groups uniformly, while *T. chilonis* struggled with fully covered egg masses. Parasitism percentage decreased with the age of the host eggs in both parasitoid species. *T. remus* showed a higher adult emergence percentage, regardless of egg scale covering but declined with increasing host egg age. The percentage of female progeny and development period were similar for both parasitoid species but decreased as egg density and egg age increased.

## 1 Introduction

The fall armyworm (FAW), *Spodoptera frugiperda* (J.E. Smith, 1797), is an invasive pest species native to tropical and subtropical regions of the America (1). Until 2015, FAW restricted to its native range (2). Its outbreak was first recorded in Africa in early 2016 (1) and in India in 2018 (3). Then, it was officially documented in Nepal in 2019 in the Nawalpur district (4). This polyphagous and devastating nature of FAW threatens maize production systems and national food security (5). In Nepal, FAW has the potential to cause 20-25% damage to maize, resulting in a loss of approximately 0.5 million tons of total maize production, valued at around 200 million dollars (6). Given the conducive climatic conditions, uncontrolled FAW infestation could completely decimate maize crops (7). Various methods exist to tackle the FAW problems in maize at farmers level with major dominance of chemical measures (8, 9). Their overuse has adverse effect on the environment, non-target organisms, and human health (10). Moreover, it triggers problems like resistance development, resurgence of pests, and the destruction of natural enemies (11). Thus, there is a global need for environmentally friendly alternatives to address the FAW problem without harming the environment and human health (12).

Biological control is an eco-friendly approach to manage FAW (13). Being a self-propelling and self-perpetuating system, this method proves to be economic and sustainable in the long run (12). It contributes as an important component of the Integrated Pest Management (IPM) approach (14). This method in combination with various other control methods carries the potential to significantly decrease the amount of pesticides being used currently (15). It makes a use of predators, parasitoids, entomopathogens, and biopesticides that attack on specific pest stages (12). The use of natural enemies like parasitoids (egg and larval), predators and microorganisms, can be the best alternative to chemical insecticides for FAW suppression (2). Egg parasitoids can control pest which makes them a potential candidate in biological control (16). Besides, they can be easily mass-produced in small area using both natural and laboratory host (17). Field surveys at various parts of the world have recorded the presence of egg parasitoids (*Trichogramma* spp., and *Telenomus* spp.), larval parasitoids (*Cotesia* spp., *Charops* spp., and dipteran species), egg-larval parasitoid (*Chelonus curvimaculatus*), as well as entomopathogenic fungi (*Beauveria bassiana* and *Metarhizium anisopliae*) with proven efficiency against FAW (18–21). These biocontrol agents are easy to rear in the laboratory and suitable for field release (14, 22). Among different biological control agents, eggs parasitoids are mostly explored and used against major agricultural pests (23). Among them, *Trichogramma chilonis* Ishii, 1941 and *Telenomus remus* Nixon, 1937 are proven to be the most promising egg parasitoids for FAW management (24) and have been recorded in different parts of Nepal (25, 26).

Parasitism by egg parasitoids is influenced by various traits of host eggs (27). Egg density affects parasitism by influencing encounter rates with small to moderate densities enhancing the efficiency, whereas higher density reducing it due to competition and superparasitism (28, 29). Host egg age also influences parasitoid preference and outcomes—fresh host can lead to higher parasitism whereas older eggs with hardened chorion and developed embryo may limit parasitoids fitness (30, 31). Scale covering on FAW egg massess acts as a physical barrier for the parasitoids like *T. chilonis* having short ovipositor (24), whereas *T. remus* are known for its ability to penetrate dense, scaly and multilayered egg masses (13).

Several studies have explored the parasitism of egg parasitoids under different conditions (13, 29, 32), however, a comprehensive study of how these host egg traits—egg density, scale cover, and egg age—influence parasitism remains limited. Moreover, comparative assessments between *T. chilonis* and *T. remus* under these conditions are scarce, despite their co-existence and overlapping use in FAW biocontrol programs. In this study, we systematically assessed and compared the parasitism, adult emergence, percentage of female progeny, and developmental duration of *T. chilonis* and *T. remus* across different densities, scale covering, and age groups of FAW eggs under laboratory conditions.

## 2 Materials and methods

### 2.1 Experimental location

The experiments were conducted during the maize-growing season (May–November 2022) under controlled laboratory conditions (mean temperature 24.3 ± 0.8 °C and RH 69.3 ± 2.2%) at the Entomology Laboratory of the National Maize Research Program (NMRP), Rampur, Chitwan, Nepal (27°39′17″N, 84°21′2″E; 228 m above sea level).

### 2.2 FAW colony establishment

Late-instar *S. frugiperda* larvae were collected from pesticide-free maize fields at NMRP and reared on fresh maize leaves in transparent plastic containers (19 x 14 x 7 cm^3^) for cohort-based rearing (33). Fresh green leaves were provided every one to two days, depending on their condition, until the larvae pupated. The pupae were collected daily and transferred to separate boxes (14 × 10 × 4 cm³). This rearing process was repeated to ensure a continuous supply of pupae for adult emergence.

### 2.3 Egg collection chamber

The adults after emergence were transferred to oviposition cages (30 × 30 × 30 cm3) supplied with water-soaked cotton, honey, and young maize plants for egg laying. The next morning, maize plants with eggs on their leaves were carefully removed. Leaves with egg masses were clipped and placed in a well-ventilated box. Medium-sized multilayered egg masses (roughly 100 eggs) were then selected and categorized into three groups based on scale covering: fully covered, partially covered, and uncovered. Similarly, single-layer egg masses were counted under a microscope using a fine camel hairbrush, and batches of 20, 40, and 60 eggs were prepared. This process was repeated every day to obtain eggs of different ages for each group. Finally, the eggs were glued onto cardboard pieces (3 × 5 cm²) to prepare standardized egg cards for the experiment.

### 2.4 Laboratory rearing of egg parasitoids

A wild population of *T. remus* were collected from the NMRP maize field and a nucleus colony was established in the laboratory. FAW egg masses were randomly collected and checked for the presence of parasitoids. The unparasitized eggs yields only FAW larvae whereas, parasitized eggs produced few or no FAW larvae after 1–3 days and adult parasitoids emerged after about 7–10 days. The emerged parasitoids were supplied with a thin layer of honey as source of energy and later identified to confirm the species. To multiply them, fresh FAW egg masses (from egg collection chamber) were irradiated in a UV chamber for 15–20 minutes to halt the development of embryo. The egg cards were then exposed to the test tube containing parasitoids at a ratio of one parasitoid per 20 eggs for 24 hours, with fresh FAW eggs supplied on alternate days until the parasitoids died, as described by Tefera et al. (2019) (14). The parasitized egg masses were kept in separate test tube, and the process was repeated for 3–4 generations. The parasitized egg masses (master’s card) were prepared and refrigerated at 4–6 °C for 1–2 days at parasitoid pupal stage. These master cards were then taken out each day to make sure that freshly emerged adult parasitoids are available for research every day.

The other species of egg parasitoid, *T. chilonis* was being successfully reared in the entomological division of NMRP, Rampur. Tricho-master cards (card with glued eggs of *Corcyra cephalonica* (Stainton, 1866) already parasitized by *T. chilonis*) were used to parasitize few fresh batches of FAW eggs. The parasitized egg masses were then used to parasitized and multiply other batches of eggs as explained for *T. remus* and after 3–4 generations, multiple batches were made and refrigerated to ensure a continuous supply of new parasitoid every day in the laboratory.

### 2.5 Parasitism at different egg densities and ages (Experiment 1)

In nature, egg parasitoids come across FAW eggs in different numbers and at different ages. The number of eggs depends on the age of the female FAW—newly mated females lay many eggs, while older ones lay fewer. FAW eggs also hatch in about 3 days, so their age varies depending on the oviposition time. To study how these differences in egg number and age affect parasitism, two egg parasitoids—*T. chilonis* and *T. remus*—were tested on FAW eggs of three different densities (20, 40, and 60 eggs) and three age groups (less than 12 hours, 24–36 hours, and 48–60 hours old). The experiment was set up using a completely randomized design (CRD) with three replications. For each test, a one-day-old mated female parasitoid was placed inside a glass test tube (15 mm × 100 mm). Data were collected on the proportion of parasitism, viable adult emergence, sex ratio (percentage of female progeny), and the developmental period of both parasitoid species.

### 2.6 Parasitism at different egg scale covering and ages (Experiment 2)

Newly mated female lays egg mass with fully covered scale and, the proportion of scale decreases and old female lays eggs without scale covering. To study how these differences in egg scale covering and age affect parasitism, *T. chilonis* and *T. remus* were evaluated on FAW eggs of three different scale covering (Fully covered, partially covered, and uncovered) and three age groups (less than 12 hours, 24–36 hours, and 48–60 hours old). The experiment was also set up using a completely randomized design (CRD) with three replications. Uniform sized egg masses (nearly 100 ± 10) were kept in a test tube and a one-day-old mated female parasitoid was placed inside a glass test tube (15 mm × 100 mm). Data were collected on the proportion of parasitism, viable adult emergence, sex ratio (percentage of female progeny), and the developmental period of both parasitoid species.

### 2.7 Statistical analysis

All data were entered into MS Excel (Office 365) for basic analysis and graph generation. Normality and homogeneity were tested using the GVLMA package, and data not meeting ANOVA assumptions were arcsine-transformed. ANOVA was conducted in RStudio (v2022.03.1), and significant results were separated using Tukey’s HSD at a 5% significance level. Univariate outliers present on the datasets were identified using box and whisker plot techniques. The influential outliers compromising and questioning the credibility of the result were handled by the variable deletion techniques i.e., data exclusion as explained by Mowbray et al. (2019) (34). Data on percentage emergence of parasitoids, female progeny percentage and developmental period for experiment 2 were compromised because of the undefined value of *T. chilonis* on one variable (fully covered). The variable was excluded for the unbiased estimate of those parameters.

## 3 Results

### 3.1 Parasitism across egg densities and host egg age

#### 3.1.1 Parasitism

Parasitism was significantly influenced by the parasitoid species, host egg density and egg age (**Table 1**). Across all egg densities and egg ages, *T. remus* parasitized 75.8% of FAW eggs, which was statistically higher than *T. chilonis*, which parasitized 63.9% of the FAW eggs (F_1,36_ = 72.49; p < 0.05). Parasitism was also influenced by egg density. Egg masses with a density of 20 eggs had the highest parasitism (81.7%), which was significantly higher than those with 40 eggs (68.8%) and 60 eggs (59.3%) (F_2_,_36_ = 91.29; p < 0.05). Host egg age directly affected parasitism, regardless of the parasitoid species. Eggs less than 12 hours old had significantly higher parasitism (83.24%), followed by eggs aged 24–36 hours (69.8%), and 48–60 hours (56.6%) (F_2_,_36_ = 121.04; p < 0.05).

**Table 1:** Effect of parasitoid species, FAW egg density and egg age on parasitism, viable parasitoid emergence and developmental duration of parasitoids.

Treatments	Parasitism (%)	Viable parasitoid emergence (%)	Developmental duration (days)
Parasitoids			
*Trichogramma chilonis*	63.95^b^ ± 3.23 (53.72)	69.52^b^ ± 3.24 (57.21)	9.8 ± 0.1
*Telenomus remus*	75.83^a^ ± 2.65 (62.12)	81.05^a^ ± 2.16 (65.53)	9.7 ± 0.1
SEM (±)	0.70	1.34	0.09
MSD	2.00	3.83	0.26
F-test	72.49^*^	19.42^*^	0.08 ^NS^
Egg density			
Twenty eggs	81.67^a^ ± 2.89 (66.71)	84.97^a^ ± 1.96 (68.38)	9.8 ± 0.1
Forty eggs	68.75^b^ ± 3.14 (56.48)	75.28^b^ ± 3.33 (61.10)	9.9 ± 0.1
Sixty eggs	59.26^c^ ± 3.64 (50.58)	65.60^c^ ± 3.81 (54.77)	9.6 ± 0.1
SEm (±)	0.85	1.64	0.11
MSD	2.95	5.66	0.39
F-test	91.29^*^	17.65^*^	1.58^NS^
Egg age			
0–12 hours old	83.24^a ±^2.49 (67.68)	85.03^a^ ± 1.82 (69.93)	9.9 ± 0.1
24–36 hours old	69.81^b^ ± 3.01 (57.15)	72.18^b^ ± 4.25 (59.90)	9.8 ± 0.1
48–60 hours old	56.62^c^ ± 3.20 (48.93)	68.65^b^ ± 3.22 (56.41)	9.6 ± 0.1
SEm (±)	0.85	1.64	0.11
MSD	2.95	5.66	0.39
F-test	121.04^*^	12.73^*^	2.58^NS^
CV (%)	6.26	11.31	4.83
Grand mean	69.89	75.28	9.76

Means ± standard error followed by the same letter in the column do not differ according to Tukey test (α, 0.05); MSD, Minimum significant difference; CV (%), Coefficient of variation. Data in parenthesis are arcsine transformed values.

#### 3.1.2 Viable parasitoid adult emergence

The percentage of viable parasitoid adult emergence was significantly influenced by the parasitoid species, egg density, and the egg age (**Table 1**). *T. remus* showed higher adult emergence (81.1%) compared to *T. chilonis* (69.5%) (F_1,36_ = 19.42; p < 0.05). Emergence percentages were highest in egg masses of 20-egg density (84.9%), followed by 40-egg density (75.3%) and 60-eggs (65.6%) (F_2,36_ = 17.65; p < 0.05). Similarly, emergence was significantly higher in eggs less than 12 hours old (85.03%), followed by eggs aged 24–36 hours (72.18%) and 48–60 hours (68.65%) (F_2,36_ = 12.73; p < 0.05).

#### 3.1.3 Sex ratio (Percentage of female progeny)

**Figure 1:**
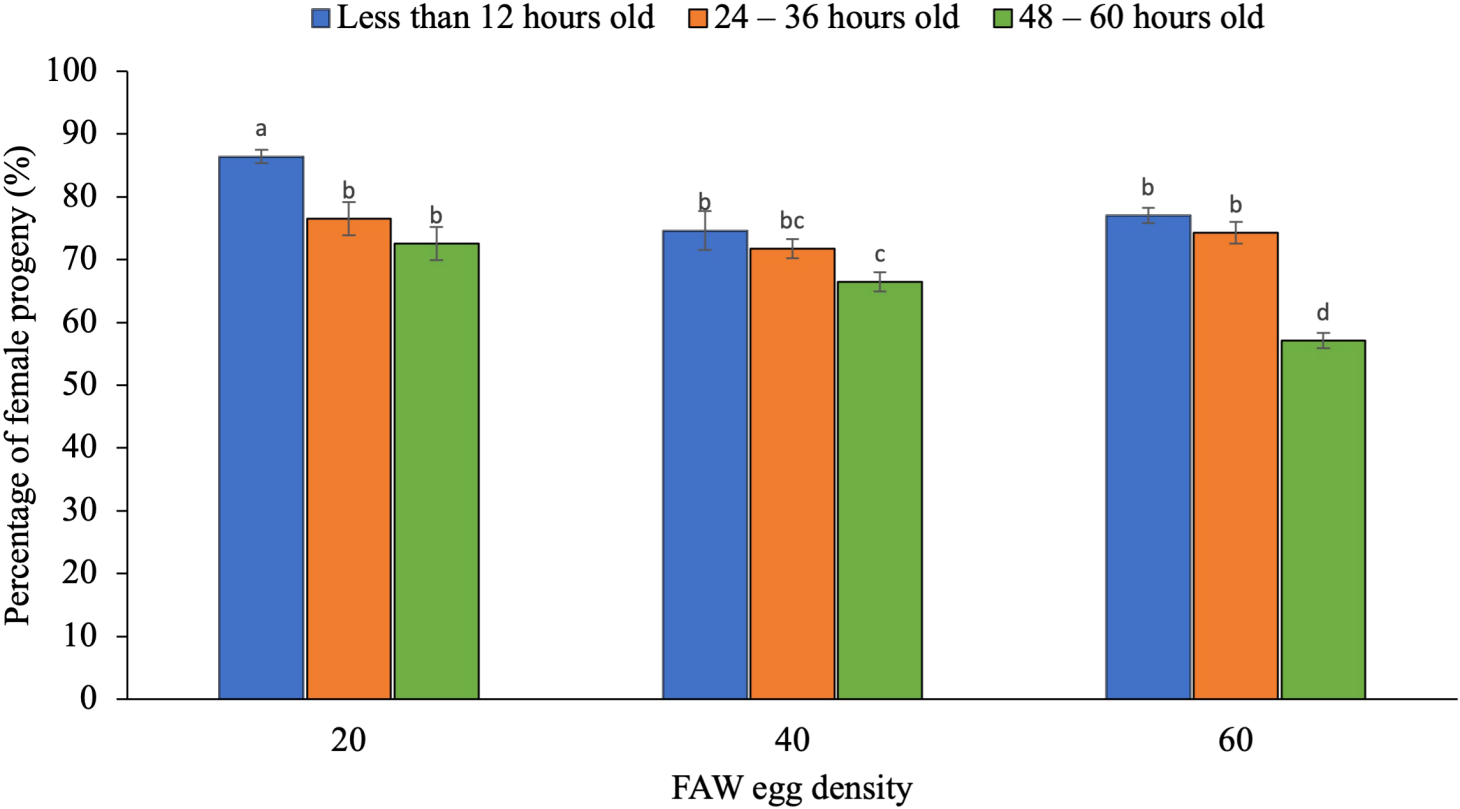
Percentage of female progeny of egg parasitoids as influenced by interaction between host egg density and egg age. The standard error bar with the same letter (s) over the bar are not significantly different from each other (Tukey HSD test, α = 0.05)

The average percentage of emerged female parasitoids in *T. chilonis* (72.73%) was statistically similar to that of *T remus* (73.20%) (F_1,36_ = 0.19; p = 0.67). The percentage of female was significantly influenced by the interaction between egg density and egg age (**Figure 1**; F_4,36_ = 3.78; p = 0.01).

#### 3.1.4 Developmental period

The developmental duration of the parasitoids was not significantly affected by parasitoid species, egg density, or egg age (**Table 1**). The developmental period of *T. chilonis* was 9.8 days and 9.7 days for *T. remus* (F_1,36_ = 0.08; p = 0.77). For both species, adult emerged after 9.8 days from 20-egg density, 9.9 days from 40-egg density and 9.6 days from 60-egg density (F_2,36_ = 1.58; p = 0.22). Parasitoids emerged after 9.9 days from less than 12 hours old egg FAW eggs, 9.8 days from 24–36 hours old eggs and 9.6 days from 48–60 hours old eggs (F_2,36_ = 2.58; p = 0.09).

### 3.2 Parasitism across egg scale covering and host egg age

#### 3.2.1 Parasitism

**Figure 2:**
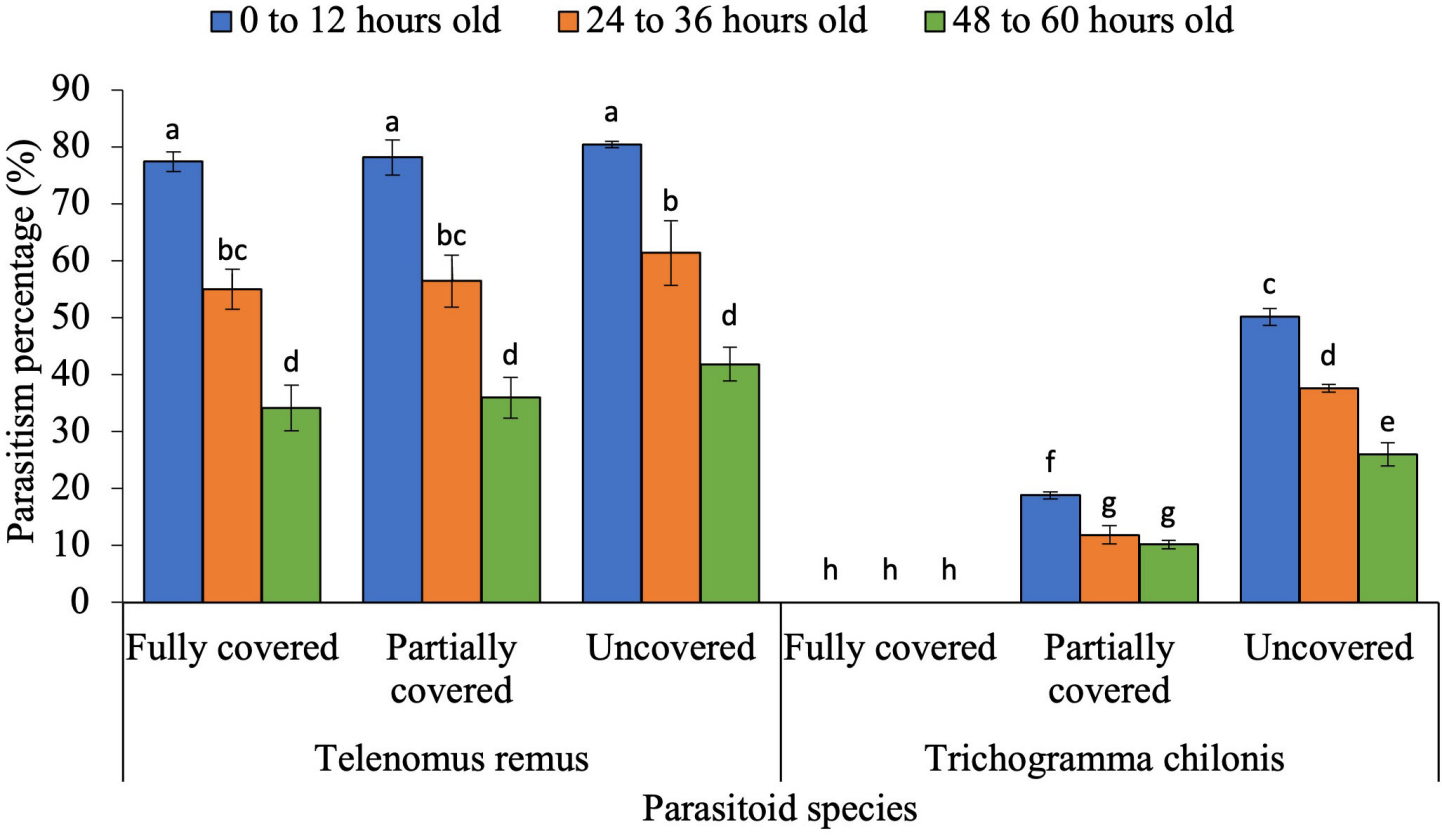
Parasitism percentage of egg parasitoids as influenced by interaction between parasitoid species and host egg age. The standard error bar with the same letter (s) over the bar are not significantly different from each other based on (Tukey HSD test, α = 0.05).

**Figure 3:**
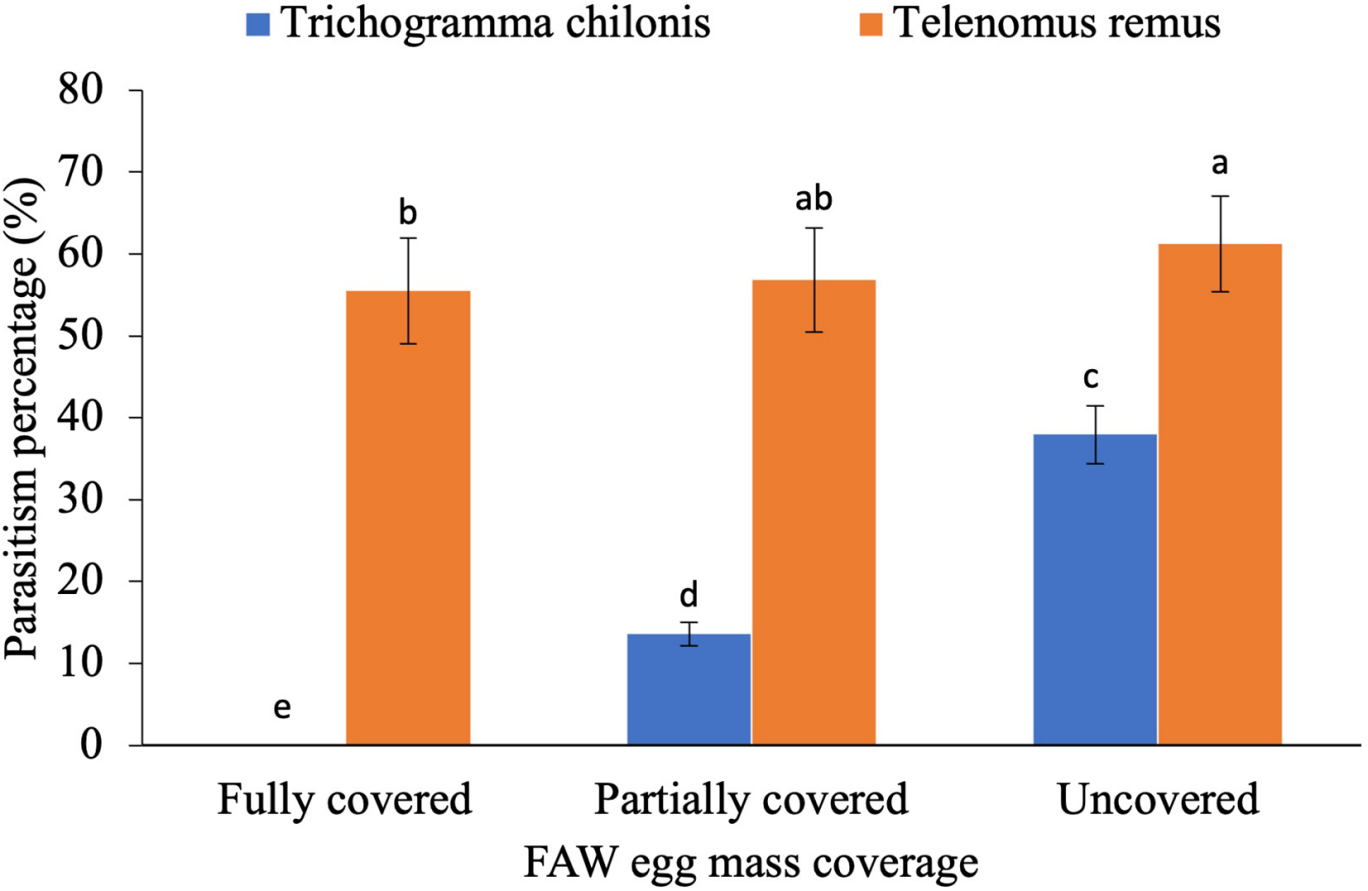
Parasitism percentage of egg parasitoids as influenced by interaction between parasitoid species and host egg scale covering. The standard error bar with the same letter (s) over the bar are not significantly different from each other (Tukey HSD test, α = 0.05).

**Figure 4:**
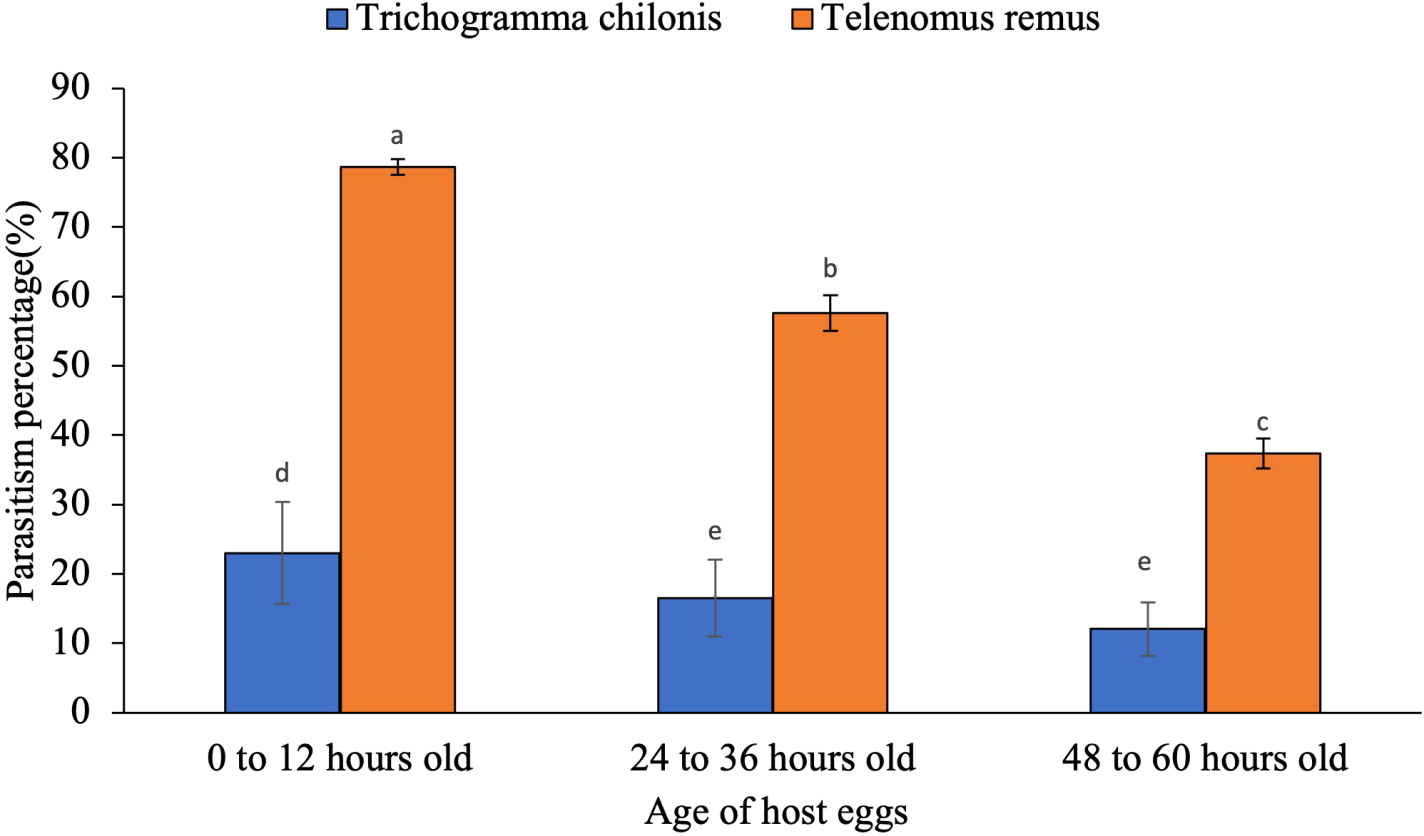
Parasitism percentage of egg parasitoids as influenced by interaction between parasitoid species, host egg scale covering and host egg age. The standard error bar with the same letter (s) over the bar are not significantly different from each other (Tukey HSD test, α = 0.05).

Parasitism was significantly influenced by the three-way interaction among the parasitoid species, host egg scale coverage, and host egg age (**Figure 2**; F_4,36_ = 3.56, p = 0.02). The parasitism percentage was also affected by the interaction between parasitoid species and host egg scale covering (**Figure 3**; F_2,36_ = 160.46, p < 0.001), as well as between parasitoid species and egg age (**Figure 4**; F_2,36_ = 43.06, p < 0.05).

#### 3.2.2 Viable parasitoid adult emergence

**Figure 5:**
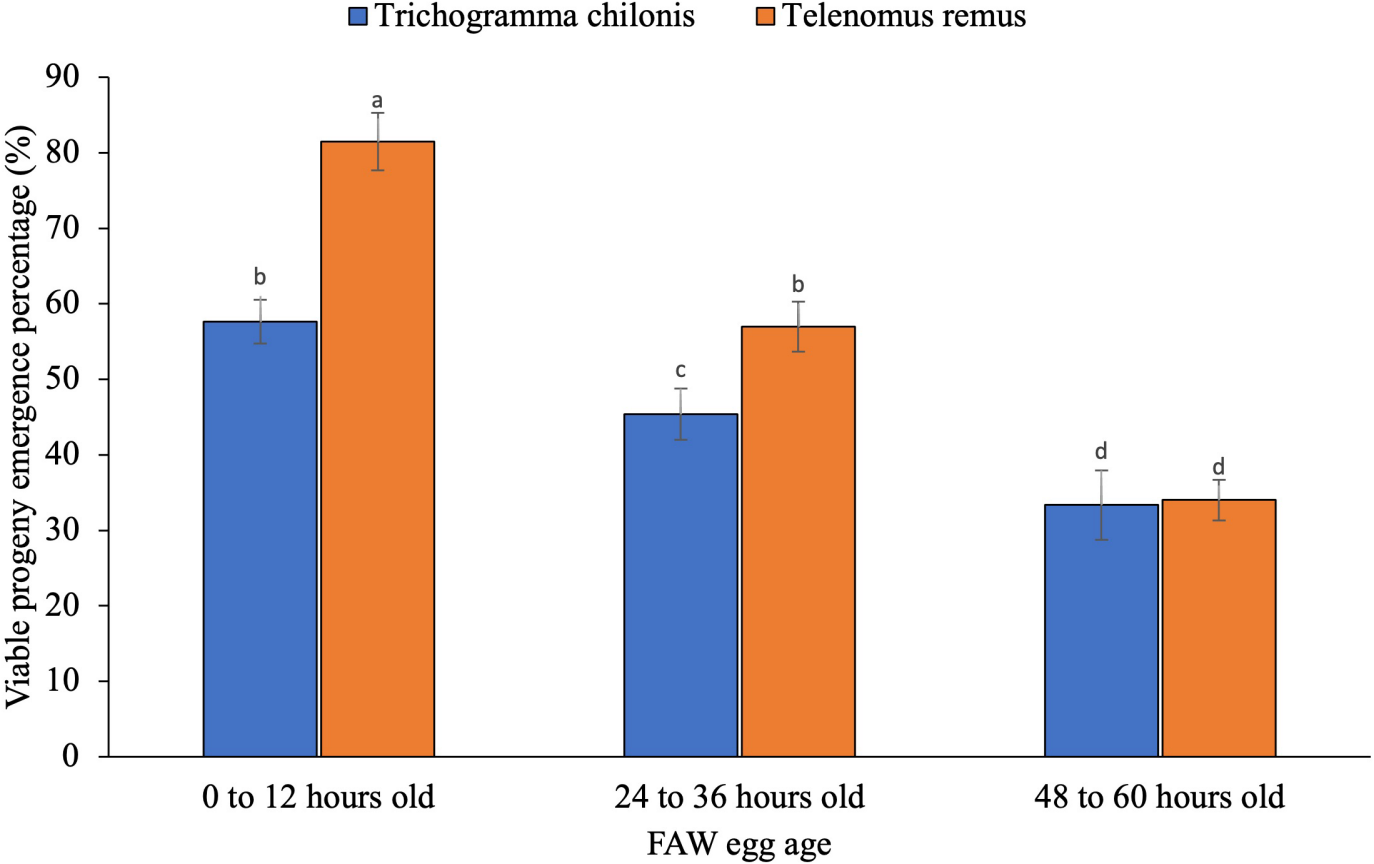
Viable parasitoid adult emergence percentage as influenced by interaction between parasitoid species, and host egg age. The standard error bar with the same letter (s) over the bar are not significantly different from each other (Tukey HSD test, α = 0.05).

The percentage of viable adult emergence was significantly influenced by the interaction between parasitoid species and host egg age (**Figure 5**; F_2,24_ = 6.22; p = 0.007) but not influenced by the egg scale covering (F_1,24_ = 3.34; p = 0.08).

#### 3.2.3 Sex ratio (Percentage of female progeny)

The percentage of emerged female was 73.19% for *T. remus* and 70.56% for *T. chilonis*, with no significant difference (**Table 2**) (F_1,24_ = 2.85; p = 0.10). Similarly, the percentage of emerged female was 72.66% in partially covered egg masses and 71.08% in uncovered egg masses for both parasitoid species, which were also statistically similar (F_1,24_ = 0.81; p = 0.38). However, egg age positively affected the emerged female. Eggs less than 12 hours old (75.49%) and 24–36 hours old (74.83%) produced significantly higher females than 48–60 hours old eggs (65.30%) (F_2_,_24_ = 16.10; p < 0.05).

**Table 2:** Effect of parasitoid species, fall armyworm egg scale covering and egg age on percentage of female progeny and developmental duration of parasitoids.

Treatments	Percentage of female progeny	Developmental duration (days)
Parasitoids		
*Trichogramma chilonis*	70.56 ± 1.46 (57.25)	9.89 ± 0.18
*Telenomus remus*	73.18 ± 1.69 (58.99)	9.72 ± 0.11
SEm (±)	0.73	0.15
MSD	2.13	0.44
F-test	2.85^NS^	0.60^NS^
Scale covering		
Fully covered	–	–
Partially covered	72.66 ± 1.33 (58.57)	9.67 ± 0.16
Uncovered	71.08 ± 1.83 (57.65)	9.94 ± 0.13
SEm (±)	0.73	0.15
MSD	2.13	0.44
F-test	0.81 ^NS^	1.67^NS^
Egg age		
0–12 hours old	75.49^a^ ± 1.44 (60.43)	9.83 ± 0.21
24–36 hours old	74.83^a^ ± 0.93 (59.93)	9.91 ± 0.19
48–60 hours old	65.30^b^ ± 1.79 (53.99)	9.67 ± 0.14
SEm (±)	0.89	0.19
MSD	2.61	0.54
F-test	16.1^*^	0.47^NS^
CV (%)	5.32	6.58
Grand mean	71.87	9.80

Means ± standard error followed by the same letter in the column do not differ according to Tukey test (α, 0.05); MSD, Minimum significant difference; CV (%), Coefficient of variation. Data in parenthesis are arcsine transformed values; ‘-’, denotes the univariate outlier data excluded before analysis to maintain validity of the result.

#### 3.2.4 Developmental period

There was no significant influence of parasitoid species, FAW egg mass coverage and ages on developmental duration of parasitoids (**Table 2**). The developmental days of *T. chilonis* was 9.9 days and for *T. remus* was 9.7 days. (F_1,24_ = 0.60; p = 0.45). Similarly, the developmental duration was 9.9 days for uncovered eggs and 9.7 days for partially covered eggs for both parasitoid species (F_1,24_ = 1.67; p = 0.21). Likewise, parasitoids emerge after 9.8 days in less than 12 hours old eggs, 9.9 days in 24–36 hours old eggs, and 9.7 days in 48–60 hours old eggs (F_2,24_ = 0.47; p = 0.63).

## 4 Discussion

### 4.1 Parasitism

This study aimed to evaluate the parasitism efficiency of *T. remus* and *T. chilonis* on *S. frugiperda* egg masses of varying structures and ages. Our finding demonstrated that *T. remus* exhibited significantly higher parasitism in both single and multilayered egg masses. Parasitism was higher for *T. remus* than *T. chilonis* in both single and multilayered egg masses, with the highest rates in single layered egg mass due to easier accessibility for parasitoids (28). The parasitism declines with egg mass layers and scale covering, reflecting the physical challenge of overcoming scales and penetrating deeper layers. These findings are supported by Beserra and Parra (2005) (28), Jin et al. (2021) (29), Mohamed (2021) (35) and Mohamed et al. (2023) (36), all of them confirmed the parasitism rates by different Trichogramma species decreased based on the number of egg mass layer.

The superior performance of *T. remus* is largely due to its robust and aggressive nature enabling it to overcome the physical barrier of scale coverings and penetrate deeper into multilayered egg masses (13). In contrast, *T. chilonis* was hindered by the scale covering, restricting it to parasitize only the upper layers or eggs on the edges (24, 30).

Parasitism decreased with increasing host egg age due to depletion of nutrient and host embryo development, which rendered older eggs unsuitable for parasitoid development (31). Also, the egg scale covering harden with age, making it more challenging for parasitoids to penetrate through it (37). Although these results demonstrate *T. remus’s* superiority over *T. chilonis* as a biocontrol agent, field complexities may not be fully captured by laboratory conditions. Future studies should evaluate their parasitic performance under natural conditions and investigate combined control methods that use both parasitoids.

### 4.2 Parasitoid adult emergence

This study investigated the emergence success of *T. remus* and *T. chilonis* from *S. frugiperda* egg masses of varying structures and ages. The results revealed significantly higher adult emergence percentage for *T. remus* compared to *T. chilonis*, which may be due to its stronger adaptation to *S. frugiperda* eggs. These findings are supported by Carneiro and Fernandes (2012) (24), who also documented higher emergence rate of *T. remus than T. pretiosum* on similar host systems.

Parasitoids emergence is influenced by host egg age. As host egg age increased, emergence rates declined, indicating that older eggs likely became less suitable for parasitoid development. This is likely due to nutrient depletion and progressive host embryo development in older eggs, which fail to support parasitoid growth and lead to higher mortality before emergence —a pattern also observed by Tuncbilek and Ayvaz (2003) (31). The findings are also supported by Priyanka et al. (2023) (32), who reported a decrease in parasitoid emergence with increasing host egg age. The emergence rates of both species were not significantly impacted by egg scale covering, suggesting this physical barrier does not hinder larval development after oviposition. This finding is supported by Laminou et al. (2020) (13) and Mohamed et al. (2023), who reported no effects of FAW egg scale coverage on emergence of egg parasitoids. These results imply that fresh or young host eggs are suitable for mass rearing of parasitoids. Additionally, *T. remus* seems to be a good option for field release against *S. frugiperda*, particularly in cases where host egg age varies. Future research should validate these findings in the field and look into possible explanations for the developmental advantages of *T. remus* offspring over those of other species.

### 4.3 Sex ratio (Percentage of female progeny)

This study investigated the influence of host egg density, egg scale covering and egg age on the percentage of female progeny in two egg parasitoids, *T. chilonis* and *T. remus*. Our study reveals that the percentage of female progeny in both species of egg parasitoids are statistically similar. This finding is in consistent with the finding of Jin et al. (2021) (29) and Chen et al. (2021) (38), who too reported the average percentage of female progeny in *T. chilonis* and *T. remus* around 70 percent.

The proportion of female offspring decreased with increasing host egg densities. This might be explained by the fact that more unfertilized *S. frugiperda* eggs were parasitized in high-density environments, which probably produced more male progeny (29). Additionally, the percentage of female progeny declined with increasing egg age. This may be possibly due to declining egg quality and reduced resources in older eggs, which negatively impact the development of female parasitoids. This is supported by findings from Sun et al. (2021) (30), who observed a drop in *Trichogramma* female progeny percentage from 82% to 69% as egg age increased from 0 to 2 days. Similarly, Priyanka et al. (2023) (32) noted a reduction in *T. remus* female progeny from 66% to 64% as egg age increased from 24 to 48 hours. Our results demonstrate that the sex ratio of emerging parasitoids is significantly influenced by the age and density of the host egg. Because more females can increase the success of parasitism, this is beneficial for mass-rearing and biocontrol initiatives. These effects should be tested in other host-parasitoid systems and in the field.

### 4.4 Developmental period of parasitoids

This study investigated the influence of host egg density, egg scale covering and egg age on the developmental duration of two egg parasitoids, *T. chilonis* and *T. remus*. Our finding reveals that the developmental time from egg to adult remained consistent across both *T. chilonis* and *T. remus*, regardless of treatment conditions. This is supported by findings of Sultan et al. (2013) (39) who reported a development period of 9.60 days for *T. chilonis*, while Oktaviani et al. (2021) (40), observed a similar duration of 9.61 days for *T. remus*.

The reason behind this stability in developmental time may lie in the biology of the parasitoids. As noted by Bueno et al. (2008) (41) these parasitoid species lay their eggs singly inside individual FAW eggs and superparasitism or multiple parasitism are rare (42). Therefore, each parasitoid larva develops independently within a single host egg, external factors such as egg density or the presence of scale covering do not appear to influence their development. Moreover, our findings are consistent with Priyanka et al. (2023) (32), who recorded developmental durations of *T. remus* as 9.61 days on 24-hour-old eggs and 9.52 days on 48-hour-old eggs. These results further support the conclusion that the age of the host egg does not significantly impact the time required for parasitoid development. Although the developmental duration of these two parasitoids is consistent across different egg traits, the small sample size and laboratory setting necessitate larger field-based research to validate and generalize the findings.

## 5 Conclusions

The higher parasitism of *T. remus* on multilayered and scaly egg masses of *S. frugiperda* indicates its potential as biological control agent for scaly egg masses. However, *T. chilonis* fails to overcome the layers and scales barrier of eggs of *S. frugiperda*. The parasitism of both parasitoid species decreases with the increasing age of host eggs, highlighting the importance of synchronization in augmentative biological control programs. For both species, laboratory mass production should coincide with the freshly laid fall armyworm eggs (within 24 hours) for efficient parasitism and sustainable female populations in the environment.

However, this study was conducted in lab condition using single female in single tube setup, which may not fully represent actual field situation. In field condition, parasitism is shaped by competition, host searching behavior, and heterogenous egg masses all of which can change parasitism compared to laboratory conditions. In addition, host egg scale coverage was measured qualitatively (fully covered, partially covered, uncovered) rather than quantitatively. Because “partial” versus “full” coverage can be subjective, and scale thickness may vary among egg masses, future studies should quantify scale coverage and thickness and validate parasitoid performance under semi-field and field conditions.

## Data Availability

The original contributions presented in the study are included in the article/**Supplementary Material**. Further inquiries can be directed to the corresponding author/s.
